# The Effect of Medication Adherence and Spirituality in Quality of Life of Patients with Rheumatic Diseases

**DOI:** 10.3390/healthcare13040436

**Published:** 2025-02-18

**Authors:** Maria Kavvadia, Maria Saridi, Aikaterini Toska, Vissarion Bakalis, Stella Zetta, Theodosios Paralikas, Pavlos Sarafis, Evangelos C. Fradelos

**Affiliations:** 1 School of Social Science, Hellenic Open University, 26335 Patra, Greece; std518821@ac.eap.gr (M.K.); saridi@uth.gr (M.S.); ktoska@uth.gr (A.T.); 2 Laboratory of Clinical Nursing, Department of Nursing, University of Thessaly, 41500 Larissa, Greece; szetta@uth.gr; 3 Department of Nursing, University of Thessaly, 41500 Larissa, Greece; bissarion_bakalis@yahoo.gr (V.B.); paralikas@uth.gr (T.P.); psarafis@uth.gr (P.S.)

**Keywords:** quality of life, spirituality, medication adherence, rheumatic diseases

## Abstract

**Background**: Spirituality is significantly associated with the quality of life of patients suffering from rheumatic diseases, helping them to cope with pain and improve emotional well-being. There is a gap in the literature regarding the relationship between spirituality, quality of life and treatment adherence in patients with rheumatic diseases, especially rheumatoid arthritis (RA) and systemic lupus erythematosus (SLE), as relevant studies, especially in the Greek population, are limited. **Aims**: The present study aimed to evaluate the effect of medication adherence and spirituality on the quality of life of patients with rheumatic diseases. **Methods**: This was a cross-sectional study conducted in adult patients with rheumatoid arthritis and systemic lupus erythematosus from the region of Crete. Data were collected via a self-administrated questionnaire consisting of four parts, including questions regarding demographic and clinical information; the WHOQoL-BREF, 3, FACIT-Sp-12, and SMAQ questionnaires were used. The level of statistical significance was set at α = 0.05 for all analyses. **Results**: The study sample consisted of 115 participants, with the majority being women (90%). The majority of the participants suffered from RA (62%), while 38% suffered from SLE. The mean age of the participants was 49.15 years (SD = 11.7), and 46% described their health as good. We found that the dimensions of spirituality are positively related to the dimensions of quality of life. The peace dimension has a strong correlation with psychological health (r = 0.679, *p* < 0.001) and overall quality of life (QOL Global, r = 0.671, *p* < 0.001). Meaning also shows a positive correlation with psychological health (r = 0.563, *p* < 0.001) and overall quality of life (r = 0.506, *p* < 0.001), whereas adherence to medication shows a low but positive correlation with overall spirituality (r = 0.192, *p* = 0.040). **Conclusions**: The findings support that spirituality can be a protective mechanism, improving the mental resilience and adaptability of patients. This study has the potential to contribute to the development of evidence-based guidelines for the integration of spiritual care into clinical practice, with the objective of enhancing the psychological well-being and overall quality of life of patients with rheumatic diseases.

## 1. Introduction

Spirituality is a multidimensional and fundamental pillar of human existence, including the search for meaning; a sense of connection with oneself, others, and the environment; and a transcendent force or divine element as perceived by everyone [1]. The concept of spirituality differs from religiosity as the latter is limited to organized religious denominations, while spirituality is a broader concept that includes values, meaning, and personal development [2]. In recent years, spirituality has been recognized as an important factor influencing health and quality of life, particularly in people suffering from chronic conditions.

Rheumatoid arthritis (RA) and systemic lupus erythematosus (SLE) are autoimmune rheumatic diseases that mainly affect the musculoskeletal system but also other organs, causing severe inflammation, stiffness, pain, and fatigue [3]. These chronic conditions bring about significant psychosocial effects, such as depression, anxiety, and reduced functioning, which limit patients’ quality of life [4]. According to studies, women are more prone to autoimmune diseases, as they make up 78% of diagnosed cases [5].

Quality of life (QoL) is a holistic concept that includes physical, psychological, social, and environmental well-being [6]. Especially for patients with chronic conditions, quality of life is not just limited to the absence of pain or symptoms but includes the ability to adapt to the challenges of the disease [7]. Health-related quality of life (HRQoL) focuses on the individual’s functioning and subjective well-being during the course of the disease [8]. In this context, spirituality can act as a protective mechanism, enhancing patients’ mental resilience and adaptability [2].

Research shows that spirituality can reduce levels of anxiety and depression, offering a sense of inner peace and meaning in life [2,9]. Inner peace and meaningfulness, as subcategories of spirituality, are positively related to all dimensions of quality of life, especially psychological and social well-being [2]. On the contrary, religious faith shows a more limited correlation with quality of life, mainly affecting the psychological dimension.

Treatment adherence is critical to the management of rheumatic diseases, as effective treatment requires patients to consistently follow medical instructions and adopt a healthy lifestyle [10]. Although the relationship between spirituality and treatment adherence is not entirely clear, spirituality may strengthen patients’ will to care for themselves and maintain a positive attitude toward their treatment [1].

Modern Orthodox spirituality in Greece is directly linked to health, as many believers seek it as a means of mental and physical empowerment. Prayer, confession, and Holy Communion are considered sources of inner peace, reducing stress and contributing to psychological well-being [11]. At the same time, fasting has not only a spiritual dimension but also beneficial effects on physical health, such as improving metabolism and cardiovascular function [12]. In addition, recourse to pilgrimages and miraculous icons, such as the Virgin Mary in Tinos, is often accompanied by hope for healing, highlighting the close relationship between faith, spirituality, and health in modern Greek society [13].

Spirituality plays an important role in the treatment of chronic diseases, as it is associated with improving mental resilience, reducing stress, and enhancing patients’ quality of life [14]. In conditions such as RA and SLE, which are characterized by chronic pain, fatigue, and psychological burden, spirituality can act as a coping mechanism, offering patients hope and a sense of meaning [15,16].

Spirituality appears to play an important role in the quality of life of patients with systemic lupus erythematosus (SLE). According to a study by Nugraha et al. [17], perceived social and spiritual support is associated with lower levels of depression and better quality of life. Social support directly affects quality of life and mental health, while spiritual support works through reducing depression, acting as a protective mechanism against the emotional burden of chronic illness. Patients who perceive support from God or a higher being show greater mental resilience, which can improve their quality of life [18].

Studies show that spirituality is significantly associated with the quality of life of patients suffering from rheumatic diseases such as RA and ankylosing spondylitis (AS). Specifically, patients with RA and AS show moderate levels of spiritual well-being, which is positively related to psychological and social quality of life, although there is no strong correlation with disease activity [19]. Spirituality helps to cope with pain and improve emotional well-being, as patients who use positive religious and spiritual coping strategies report higher levels of positive attitude and lower levels of negative attitude [20]. Also, spirituality acts as a protective mechanism against psychological stress, providing mental resilience and improving social support [20]. Compared to AS patients, RA patients show lower levels of spiritual well-being, yet faith remains strong in RA patients [19]. Daily spiritual experiences, such as feeling connected to a higher power or seeking inner peace, are associated with better pain management and improved quality of life [20]. Although mental well-being is not directly related to disease severity, it is a critical parameter in the holistic care of patients with chronic rheumatic diseases [21]. The present study is necessary to understand the relationship between spirituality, quality of life, and treatment adherence in patients with RA and SLE. Although there is a lot of research on the role of spirituality in chronic diseases, studies focusing on rheumatic diseases are limited, especially in the Greek population [2,21,22,23]. Investigating these factors may provide valuable information for healthcare professionals to incorporate the spiritual dimension into the holistic care of patients with rheumatic diseases. By better understanding the role of spirituality, interventions can be developed that improve quality of life and support treatment adherence.

The necessity and importance of this study are that it offers a holistic approach to the management of chronic autoimmune diseases, integrating the spiritual dimension into clinical practice. In this way, healthcare professionals can provide more comprehensive care, improving not only physical symptoms but also patients’ psychological and social well-being. The purpose of the present study was to investigate the contribution of spirituality to the quality of life of patients with rheumatic diseases, specifically in patients with RA and SLE. This study seeks to answer critical research questions, such as differences in spirituality and quality of life between the two diseases, the relationship of spirituality to treatment adherence, and the effect of health and treatment on overall quality of life.

## 2. Materials and Methods

### 2.1. Study Design

This study was designed as a descriptive study of correlation, a contemporary type (cross-sectional study). The target population was adult patients with RA and SLE from the region of Crete. The study sample consisted of members of an association of patients with rheumatic diseases in Crete. The sample of 115 people was selected through random sampling and was considered representative of the specific population group. Men and women with diagnosed rheumatic diseases, who were not in an acute phase of the disease, participated.

### 2.2. Data Collection Tools

The questionnaire used consists of three parts:

1. Demographic information: includes age, gender, duration of illness, and other relevant information.
2. WHOQoL-BREF: The World Health Organization quality of life questionnaire (WHOQoL-BREF), adapted to Greek, examines four dimensions of quality of life: physical, mental, social, and environmental. Uses a 5-point Likert scale (1 = “not at all” to 5 = “very much”) [5,24]. In this study, the Cronbach’s alpha value for the WHOQoL-BREF was a = 0.901.
3. FACIT-Sp-12: The Spiritual Well-Being Scale of Functional Assessment in Chronic Illness Treatment (FACIT-Sp-12) consists of 12 questions that measure three dimensions: meaning-making, peace, and faith. The score ranges from 0 (“not at all”) to 4 (“very much”), with higher scores indicating higher mental well-being [25,26]. In this study, the Cronbach’s alpha value for the FACIT-Sp-12 was a = 0.801.
4. Simplified drug Adherence Questionnaire (SMAQ) is a self-reported measure that assesses medication adherence, especially useful for chronic conditions like HIV, diabetes, and hypertension. It consists of six questions about missing doses, how often they happen, dosage changes, trouble taking medicine at certain times, recent missed doses, and subjective compliance evaluation. Usually, responses are binary (yes/no), and a patient may be labeled as non-compliant based on just one negative indicator (such as missed medication or a change in dosage) [27,28]. In this study, the Cronbach’s alpha value for the SMAQ was a = 0.760.

### 2.3. Ethical Consideration

This study was conducted in accordance with the Declaration of Helsinki and approved by the Ethics Committee of the Nursing Department of University of Thessaly (38EX2024) and the committee of the Postgraduate Program “Management of Aging and Chronic Diseases” of the Hellenic Open University. Informed consent was obtained, and participants filled out the questionnaires anonymously, ensuring their confidentiality and personal data. Participation was voluntary, and participants could withdraw at any time.

### 2.4. Data Analysis

Analysis of demographic data was performed using descriptive statistics, where absolute and relative frequencies were used to capture categorical variables, such as sex, age, diagnosis, and disease duration. Means and standard deviations were calculated for continuous variables such as participant age and questionnaire scores. To investigate possible differences in questionnaire scores between diagnostic groups (RA and SLE), *t*-test for independent samples was applied. This test was used to determine whether the means of the two groups differ significantly at the level of statistical significance (α = 0.05). The value of Pearson’s coefficient (r) ranged from −1 to +1, where positive values indicate a positive correlation and negative values indicate a negative correlation. The statistical significance of correlations was assessed at a significance level of α = 0.05. In addition, to investigate the predictive ability of spirituality on overall quality of life and emotional state, linear regression was applied. The independent variables included the spirituality score (FACIT-Sp-12), and the dependent variable was the global quality of life score (WHOQoL-BREF) or emotional state score. Linear regression assessed the magnitude and statistical significance of the effect of spirituality on the other variables, with the R^2^ coefficient indicating the proportion of variance explained by the model. All statistical tests and analyses were performed with IBM SPSS software (version 26). The conditions for performing parametric analyses, such as normality of the data (Kolmogorov–Smirnov test) and homogeneity of variances (Levene’s test), were checked before the analysis. The level of statistical significance was set at α = 0.05 for all analyses.

## 3. Results

### 3.1. Descriptive Statistics

The study sample consisted of 115 participants, with the majority being women (90%). Regarding the level of education, most of the participants were tertiary education graduates (52%), and 9% held a master’s degree. Most were married (67%), 17% single, and 9% were widowed, whereas 84% of them lived with others. Regarding occupational status, 68% worked full time, 12% were retired, and 10% engaged in household activities. From a clinical point of view, 62% of the participants suffered from RA, and 38% from SLE. Regarding self-assessment of health, 46% described their health as good, 37% as neutral, and 8% as poor. The mean age of the participants was 49.15 years (SD = 11.7), with a range of 21 to 80 years. The mean disease duration was 8.1 years (SD = 8), with a range of 1 to 44 years (Table 1).

Table 2 presents the statistical results for the quality of life (QoL) and spirituality scores of patients with rheumatic diseases, as well as medication adherence. In the dimension of physical health (QOL Physical Health), values ranged from 8 to 19.1, with a mean of 13.3 and a standard deviation of 2.4, while in psychological health (QOL Psychological Health), the mean was 13.1 (SD = 2, 6). Social relationships (QOL Social Relationships) recorded a higher mean (14.2), with a standard deviation of 2.7, while the social/environmental dimension (QOL Social Environment) showed a mean of 13.6 (SD = 1.9). Overall quality of life dimensions (QOL overall G1 and QOL Health G2) showed means of 13.9 (SD = 2.0) and 13.2 (SD = 2.3), respectively. The overall quality of life score (QOL Global) ranged from 34.7 to 73.8, with a mean of 54.1 and a standard deviation of 7.6. Regarding spirituality, the dimension of meaning (FACIT Meaning) had a mean of 12.7 (SD = 3.0), while peace (FACIT Peace) had a lower mean of 8.1 (SD = 3.2). The faith dimension (FACIT Faith) had a mean of 8.3, with a larger variance (SD = 4.1). The total spirituality score (FACIT Total) ranged from 10 to 47, with a mean of 29.1 and a standard deviation of 7.6. Finally, medication adherence, as measured using the Simplified Medication Adherence Questionnaire, had a mean of 3.76 (SD = 1.2) on a scale of 0 to 5, indicating relatively good patient compliance.

### 3.2. Bivariate Analysis

#### 3.2.1. The Association Between Demographic and Clinical Characteristics with the Scales Used in This Study

Upon the examination of independent samples *t*-test, non-significant differences were observed in the scores of QoL, spirituality and medication adherence between the two groups of patients (rheumatoid arthritis and lupus). The age of the participants was found to have a significant correlation to faith (r = 0.204, *p* < 0.001) and to medication adherence score (r = 0.204, *p* < 0.001). Marital status was found to be associated with the domain of social environment on QoL (F 3,11 = 2.806, *p* = 0.043), where a significant difference was observed on the score of married (14.55 ± 2.5) and widowed (12.08 ± 1.7), t = −2.754, *p* (bonf) = 0.041.

#### 3.2.2. The Relationship Between QoL, Spirituality and Medication Adherence

Table 3 presents the results of correlation analysis of the scales used in the present study. The results of the analysis showed that the dimensions of spirituality were positively related to the dimensions of quality of life. Specifically, peace of mind (peace) had a strong correlation with psychological health (r = 0.679, *p* < 0.001) and overall quality of life (QOL Global, r = 0.671, *p* < 0.001). The meaning also showed a positive correlation with psychological health (r = 0.563, *p* < 0.001) and overall quality of life (r = 0.506, *p* < 0.001). Faith had weaker correlations, such as with psychological health (r = 0.205, *p* = 0.028) and overall quality of life (r = 0.205, *p* = 0.028). Spirituality total was positively related to psychological health (r = 0.621, *p* < 0.001) and overall quality of life (r = 0.595, *p* < 0.001). Adherence to medication showed a low but positive correlation with overall spirituality (r = 0.192, *p* = 0.040). For more detailed information, please refer to the table above with Pearson coefficients and levels of statistical significance (*p*-values).

### 3.3. Multivariable Analysis

Table 4 presents the multiple regression results, showing that peace of mind is the most powerful predictor for almost all dimensions of quality of life (QoL), with statistically significant effects on physical health (β = 0.491, *p* < 0.001), psychological health (β = 0.535, *p* < 0.001), social relationships (β = 0.434, *p* < 0.001), and the social environment (β = 0.252, *p* = 0.020). On the contrary, meaning significantly affected only psychological health (β = 0.263, *p* = 0.002), while faith did not seem to have a statistically significant effect on the quality of life dimensions. Medication adherence showed positive but not statistically significant effects on all dimensions of quality of life. The total percentage of variance explained by the models (R^2^) ranged from 11.3% for social environment to 50.4% for psychological health. For detailed results and regression coefficient values, please refer to the table above.

## 4. Discussion

The purpose of the present study was to investigate the contribution of spirituality to the quality of life of patients with rheumatic diseases, specifically in patients with RA and SLE. The results of the present study highlight the significant relationship between spirituality and quality of life in patients with rheumatic diseases, such as RA and SLE.

The present study is a pioneering approach to the evaluation of the relationship between spiritual compliance and quality of life in patients with SLE and RA in the Greek population. The importance of this research lies in its inclusion within a framework that combines spirituality with the medical approach to chronic autoimmune diseases in an Orthodox context. In Greece, modern Orthodox spirituality occupies an important place in the lives of believers and is directly linked to health and well-being, as spiritual practice, such as prayer, confession, and Holy Communion, is considered a source of mental empowerment and physical relief [29]. Greek society, with its strong religious tradition, offers a unique field of study, where spirituality often acts as a catalyst for patients’ emotional and physical resilience. At the same time, fasting, which is a central element of Orthodox practice, is linked not only to the spiritual dimension but also to strengthening physical health, improving metabolism, and the functioning of the cardiovascular system [11]. By exploring this relationship, this research contributes to enhancing our understanding of the role of spirituality in the daily lives of patients with chronic conditions, promoting the need for a multidimensional approach to health that incorporates spirituality as an equally important factor with medical treatments.

According to the results, the peace dimension has the strongest positive correlation with psychological health (r = 0.679, *p* < 0.001) and overall quality of life (r = 0.671, *p* < 0.001). This is confirmed by previous studies, such as by Bartlett et al. (2003), which involved 77 patients with RA and showed that spirituality is positively related to psychological well-being and overall quality of life in people with RA. Meaning-making and inner peace help reduce anxiety and depression, enhancing the ability to adapt to illness. In contrast, organized religious practice had less of an effect. Integrating spiritual care into clinical practice can improve holistic patient care [30]. Similarly, a study by Keefe et al. (2001) [20], which involved 35 patients with RA, highlighted the important role of daily spirituality and religious coping strategies in the management of RA. Patients who use spiritual and religious practices report higher levels of positive attitude and lower levels of negative attitude. Spirituality acts as a protective mechanism against psychological stress, contributing to better adaptation to illness and improving quality of life. Spiritual serenity is instrumental in managing anxiety and depression in patients with chronic conditions, offering a sense of inner calm and meaning. It is documented that spirituality can improve mental well-being by reducing stress and enhancing psychological resilience. The study by Harvard T.H. Chan School of Public Health found that focusing on spirituality as part of healthcare can lead to better quality of life and improved medical decisions [31].

In addition, engaging in spiritual practices such as meditation and prayer has been linked to reduced levels of anxiety and depression, providing emotional relief and a sense of meaning in patients’ lives [32]. Studies indicate that mental peace helps regulate emotions, accept difficult situations, and develop positive coping mechanisms. Integrating spiritual care into clinical practice appears to provide a more holistic approach, caring for the patient not only physically but also psychologically and spiritually [33].

According to the results of this study, there is a positive correlation of meaning with psychological health, considering the direct relationship between “meaning” and improvements in psychological well-being (r = 0.563, *p* < 0.001) and overall quality of life (r = 0.506, *p* < 0.001), which is reinforced by research highlighting the importance of meaning-making for mental health. Many studies support this connection, showing that having meaning in life can act as a protective mechanism against psychological stress. For example, a study by Aftab et al. (2019), on the relationship between meaning and well-being, shows that the “presence of meaning” is associated with better physical and mental health, while “seeking meaning” is negatively associated with mental health, especially in elderly people [34]. This study highlights that meaning in life can reduce stress and enhance emotional resilience, making it a critical factor in coping with stress, especially at older ages or during major life changes. Additionally, this research found that having meaning in life is associated with less stress and better cognitive functioning, reinforcing the notion that having meaning can be protective against the negative effects of aging and declining health [34]. This is consistent with the idea that meaning acts as an emotional “sink”, helping individuals to better manage their challenges. All the above studies reinforce the view that enhancing meaning in life can significantly improve mental health while supporting the idea that helping individuals find and cultivate meaning in their lives can improve their psychological well-being.

The faith dimension appears to have a less strong correlation with psychological health and overall quality of life. This observation is confirmed by the literature, which argues that religious belief mainly affects the psychological dimension of quality of life but with a limited impact compared to the broader concept of spirituality. For example, a study conducted by Counted et al. (2018) examines the relationship of the spiritual dimension with quality of life and concludes that relational spirituality is linked to improvements in quality of life, as it enhances mental health, social connection, and the ability of individuals to withstand psychological stress [34]. However, this spirituality operates primarily through personal and subjective parameters, rather than relying on external religious practices, indicating that religious belief, as an institutionalized system, affects the quality of life in a more limited context. A study by Saffari et al. (2017) also demonstrates that spiritual/religious practices can contribute to patients’ quality of life and health, while religiosity is associated with positive effects on mental well-being, but in the sense of spiritual functioning rather than religious traditions followed [35]. Therefore, while faith can affect mental health and quality of life, the broader concept of spirituality appears to have a stronger effect, as it incorporates dimensions such as meaning-making and the search for meaning, which act protectively against anxiety and depression. These findings are reinforced by the literature, showing that spiritual practices and relational spirituality have a more significant effect on psychological health and quality of life than traditional religiosity, which primarily affects mental well-being through religious beliefs [2,36].

Adherence to medication shows a positive, albeit weak, correlation with overall spirituality (r = 0.192, *p* = 0.040). Our findings are consistent with the general belief that spirituality can enhance patients’ willingness to take care of themselves and maintain a positive attitude toward their treatment. In a review by Badanta-Romero et al. (2018), it was found that the effect of religiosity and spirituality on adherence depends on several factors. Although there are cases of a positive association, other studies have reported reduced adherence due to conflicting religious beliefs with medication. Overall, it highlights the need for sensitivity and understanding by healthcare professionals to integrate patients’ spiritual needs into their approach [37]. Similarly, in 2022, a systematic review focused on patients with cardiovascular diseases (CVDs) showed a positive association between religiosity/spirituality (R/S) and adherence, especially through participation in religious activities or inner spirituality. However, there have also been conflicting results. The study concludes that interventions that take cultural and spiritual beliefs into account may improve adherence [35]. Finally, according to a recent study of patients with type II diabetes, spiritual well-being was negatively related to treatment adherence, while hope had a positive but inverse effect on it. Here, the importance of spirituality appeared to be more related to general psychological well-being and less to immediate compliance [38]. Spirituality appears to influence medication adherence in a variety of ways. Although a positive correlation is generally observed, variations according to the cultural context, type of illness, and religious beliefs of the patients necessitate an individualized approach. Incorporating spirituality into care can enhance adherence and therapeutic effectiveness [39].

### Limitations of the Study

This study relies on self-reported data, which may affect the accuracy of the results due to socially desirable responses. In addition, the sample mainly includes women, which limits the generalizability of the findings to the male population. Finally, the correlational nature of this study does not allow for the investigation of causal relationships.

Future research could examine the relationship between spirituality and quality of life in patients with other chronic conditions, such as cardiovascular disease or cancer, to assess whether the results are common or specific to each disease. In addition, the inclusion of diverse demographic groups, with different cultural and religious traditions, could provide valuable insights into how spirituality affects health globally. Conducting studies over a longer period, as well as investigating the mechanisms through which spirituality affects physical and mental well-being, would enhance our understanding of this relationship. Finally, the development and evaluation of interventions that combine spirituality with medical care to improve patients’ quality of life could offer practical benefits in clinical practice.

## 5. Conclusions

The present study highlighted the significant relationship between spirituality and quality of life in patients with rheumatic diseases, confirming that the spiritual dimension can act as a protective mechanism against anxiety, depression, and the psychosocial effects of the disease. Specifically, inner peace and the search for meaning in life were positively associated with psychological well-being, while faith had a more limited effect. In addition, medication adherence was found to be positively, albeit weakly, associated with spirituality, suggesting that enhancing the spiritual dimension could help improve patients’ self-care. The findings of this study support the need to integrate spiritual care into clinical practice to improve the holistic approach to the treatment of patients with rheumatic diseases. Future research could further explore the relationship between spirituality and treatment adherence, as well as evaluating interventions that enhance spiritual well-being, with the goal of improving patients’ quality of life.

## Figures and Tables

**Table 1:** Demographic and clinical characteristics of the sample (n = 115).

		Absolute Frequency	Relative Frequency
Gender	Male	12	10%
	Female	103	90%
Education	Some elementary grades	4	4%
	Elementary school	3	3%
	High school	8	7%
	Lyceum	30	26%
	Tertiary Education	60	52%
	Postgraduate title	10	9%
Marital Status	Single	19	17%
	widower	10	9%
	Married	77	67%
	Divorced	9	8%
Live Alone/With Others	Alone	19	16%
	With others	96	84%
Hours Of Employment/Employment	Full time	78	68%
	Part-time	7	6%
	Student	1	1%
	Housekeeping	12	10%
	Retired	14	12%
	Unemployed	3	3%
Disease	Rheumatoid Arthritis	71	62%
	Erythematosus lupus	44	38%
How Good Of Health You Have	Very bad	4	4%
	Bad	9	8%
	No good, no bad	43	37%
	Good	53	46%
	Very good	6	5%
	Mean	Standard Deviation	Minimum–Maximum
Age	49.15	11.7	21–80
Years Of Disease	8.1	8	1–44

**Table 2:** Descriptive statistics for QoL, spirituality and medication adherence.

	Min	Max	Mean	St. Deviation
QOL Physical Health	8	19.1	13.3	2.4
QOL Psychological Health	6	19.3	13.1	2.6
QOL Social Relationships	6.4	19.2	14.2	2.7
QOL Social Environment	8.5	18.5	13.6	1.9
QOL overall G1	8.2	18.5	13.9	2.0
QOL Health G2	7.9	18.9	13.2	2.3
QOL Global	34.7	73.8	54.1	7.6
FACIT Meaning	6	16	12.7	3.0
FACIT Peace	0	15	8.1	3.2
FACIT Faith	0	16	8.3	4.1
FACIT Total	10	47	29.1	7.6
Simplified medication Adherence Questionnaire	0	5	3.76	1.2

**Table 3:** Pearson’s correlations analysis.

Variable		Physical Health	Psychological Health	Social Relationships	Social Relationships	QOL Overall	QOL Health G2	QOL_ Global
Meaning	Pearson’s r	0.333 **	0.563 **	0.408 **	0.254 *	0.395 **	0.496 **	0.506 **
Peace	Pearson’s r	0.534 **	0.679 **	0.519 **	0.345 **	0.513 **	0.666 **	0.671 **
Faith	Pearson’s r	0.131	0.205 *	0.177	0.123	0.178	0.185 *	0.205 *
Spirituality Total	Pearson’s r	0.429 **	0.621 **	0.477 **	0.313 **	0.470 **	0.579 **	0.595 **
Medication adherence	Pearson’s r	0.065	0.144	0.150	0.151	0.172	0.116	0.161

** *p* < 0.001, * *p* < 0.05.

**Table 4:** Multiple regression analysis with QoL domains as dependent variables and spirituality and medication adherence as independent variables (adjusted for gender, age, educational level, marital status, and diagnosis).

Dependent Variable Physical Health							
						95% CI	
	Unstandardized	Standard Error	Standardized	t	*p*	Lower	Upper
(Intercept)	9.982	2.295		4.350	<0.001	5.432	14.532
Meaning	0.028	0.079	0.036	0.358	0.721	−0.128	0.185
Peace	0.360	0.071	0.491	5.098	**<0.001**	0.220	0.500
Faith	0.045	0.051	0.078	0.885	0.378	−0.056	0.147
Adherence	0.063	0.158	0.034	0.401	0.689	−0.249	0.376
F(10,104) = 5.380, *p* < 0.001, R^2^ = 27.4%							
**Dependent Variable Psychological Health**							
						95% CI	
	Unstandardized	Standard Error	Standardized	t	*p*	Lower	Upper
(Intercept)	7.735	2.106		3.672	<0.001	3.558	11.912
Meaning	0.232	0.072	0.263	3.205	**0.002**	0.088	0.375
Peace	0.436	0.065	0.535	6.720	**<0.001**	0.307	0.565
Faith	0.040	0.047	0.062	0.848	0.398	−0.053	0.133
Adherence	0.105	0.145	0.051	0.726	0.469	−0.182	0.392
F(10,104) = 12.661, *p* < 0.001, R^2^ = 50.4%							
**Dependent Variable Social Relationships**							
						95% CI	
	Unstandardized	Standard Error	Standardized	t	*p*	Lower	Upper
(Intercept)	10.088	2.587		3.900	<0.001	4.959	15.218
Meaning	0.105	0.089	0.115	1.187	0.238	−0.071	0.282
Peace	0.368	0.080	0.434	4.625	**<0.001**	0.210	0.526
Faith	0.057	0.058	0.085	0.985	0.327	−0.058	0.172
Adherence	0.191	0.178	0.090	1.073	0.286	−0.162	0.543
F(10,104) = 6.217, *p* < 0.001, R^2^ = 31.4%							
**Dependent Variable Social Environment**							
						95% CI	
	Unstandardized	Standard Error	Standardized	t	*p*	Lower	Upper
(Intercept)	10.302	2.043		5.043	<0.001	6.252	14.353
Meaning	0.042	0.070	0.066	0.601	0.549	−0.097	0.181
Peace	0.149	0.063	0.252	2.365	**0.020**	0.024	0.274
Faith	0.044	0.046	0.094	0.962	0.338	−0.047	0.134
Adherence	0.171	0.140	0.115	1.215	0.227	−0.108	0.449
F(10,104) = 2.452, *p* < 0.001, R^2^ = 11.3%							
**Overall QoL**							
						95% CI	
	Unstandardized	Standard Error	Standardized	t	*p*	Lower	Upper
(Intercept)	10.195	1.939		5.257	<0.001	6.349	14.041
Meaning	0.074	0.067	0.108	1.108	0.270	−0.058	0.206
Peace	0.259	0.060	0.411	4.330	**<0.001**	0.140	0.377
Faith	0.050	0.043	0.102	1.164	0.247	−0.035	0.136
Adherence	0.181	0.133	0.115	1.355	0.178	−0.084	0.445
F(10,104) = 5.773, *p* < 0.001, R^2^ = 29.5%							

## Data Availability

Data supporting this study are available from the corresponding author upon reasonable demand.
